# Resistance to multikinase inhibitor actions mediated by insulin like growth factor-1

**DOI:** 10.1186/s13046-015-0210-1

**Published:** 2015-09-02

**Authors:** Catia Lippolis, Maria Grazia Refolo, Rosalba D’Alessandro, Nicola Carella, Caterina Messa, Aldo Cavallini, Brian Irving Carr

**Affiliations:** Department Clinical Pathology, Laboratory of Cellular and Molecular Biology, National Institute for Digestive Diseases, IRCCS “Saverio de Bellis”, Via Turi 27, 70013 Castellana Grotte, BA Italy; Izmir Biomedicine and Genome Center, Dokuz Eylul University, Izmir, Turkey

**Keywords:** Platelets, Insulin growth factor, Regorafenib, HCC cells

## Abstract

**Background:**

Blood platelet numbers are correlated with growth and aggressiveness of several tumor types, including hepatocellular carcinoma (HCC). We previously found that platelet lysates (hPLs) both stimulated HCC cell growth and migration, and antagonized the growth-inhibitory and apoptotic effects of Regorafenib, multikinase growth inhibitor, on HCC cell lines. We evaluated the effects of human insulin-like growth factor-1 (IGF1), a mitogen contained in platelets, on the Regorafenib-mediated growth inhibition.

**Methods:**

An Elisa kit was used to evaluate hPL IGF1 concentrations. The effects of IGF1 on cell proliferation were assessed with MTT assay and analysis of cell cycle progression. Apoptosis assays, scratch assay and Transwell assay were performed to measure apoptosis, cell migration and invasion respectively. Western blots were performed by standard protocols.

**Results:**

IGF1 antagonized growth inhibition exerted by Regorafenib on HCC cell lines. Moreover the mitogen blocked Regorafenib-induced apoptosis and decreased the rate of cell migration and invasion. The IGF1 effects were in turn antagonized by actions of a potent IGF1 receptor inhibitor, GSK1838705A, showing that the IGF1 receptor was involved in the mechanisms of IGF1-mediated blocking of Regorafenib action. GSK1838705A also partially blocked the effects of hPLs in antagonizing Regorafenib-mediated growth inhibition, showing that IGF1 was an important component of hPL actions.

**Conclusions:**

These results show that IGF1 antagonized Regorafenib-mediated growth, migration and invasion inhibition, as well as the drug-mediated induction of apoptosis in HCC cells and reinforce the idea that microenvironmental factors can influence cancer drug actions.

## Introduction

Hepatocellular carcinoma (HCC) occurs most frequently in livers that have been chronically damaged by hepatitis B or C, chronic alcohol ingestion, or a wide range of chronic metabolic disturbances. Most cases develop in association with liver fibrosis or cirrhosis, especially in the presence of hepatitis C, although chronic infection with hepatitis B can lead to HCC without cirrhosis and HCC [1, 2]. A consequence of the liver fibrosis is portal hypertension, with associated splenomegaly, that can cause thrombocytopenia. The latter has been considered to be a warning sign of impending HCC development in patients with chronic virus hepatitis or cirrhosis [3–5]. Thrombocytopenia-associated HCC has recently been shown to be associated with smaller-size tumors [6, 7]. In contrast, several reports have shown that large size HCCs often have normal or elevated (thrombocytosis) platelet counts [8–13], likely due to less portal hypertension. There are multiple reports of thrombosis and thrombocytosis in various cancer types [14–20].

Platelets have been shown to be involved in tumor metastasis, as well as in HCC growth, in addition to their well-recognized role in blood coagulation [21, 22]. Human platelet lysates (hPL) are a source of many growth factors and have been recently introduced into clinical practice as an adjunct to wound healing [23–27]. We previously found that hPL have anti-apoptotic effects in HCC cells and can antagonize the apoptotic and cell growth inhibitory actions of the multikinase inhibitors, Sorafenib and Regorafenib [28, 29]. The current work extends previous findings, by showing that growth inhibitory actions mediated by Regorafenib [30] in HCC cells *in vitro* can be antagonized by insulin like growth factor 1 (IGF1), one of the well-described mitogens contained in platelets [25–27]. Furthermore, an IGF1 receptor inhibitor can partially block the drug resistance actions of hPL, supporting the idea that platelet-associated IGF1 may modulate HCC resistance to multikinase inhibitor effects.

## Materials and methods

### Cells and drugs

Regorafenib was gifts from the Bayer Corp (West Haven, CT, USA), recombinant human IGF1 was purchased from Pepro-Tech (Rocky Hill, NJ, USA), GSK1838705A was purchased from Selleckchem (Houston, TX, USA).

Hep3B, HepG2 and PLC/PRF/5 human HCC cells were purchased from the American Type Culture Collection (ATCC, Rockville, MD, USA). The culture medium was Dulbecco’s Modified Eagle’s Medium (DMEM). All cell culture components were purchased from Sigma- Aldrich (Milan, Italy).

### Cell culture

Cells were cultured in DMEM in monolayer culture, and supplemented with 10 % fetal bovine serum (FBS), 100 U/ml penicillin, 100 μg/ml streptomycin, and incubated at 37 °C in a humidified atmosphere containing 5 % CO_2_ in air.

### Platelet lysates

The hPL were blood bank time-expired bags, from healthy volunteers. The study protocol was approved by the institutional review boards of the University of Bari and “Saverio de Bellis” Institute of Castellana G. (BA), Italy. Additionally, written informed consent was obtained from participants for the use of their blood in this study. The platelet-rich plasma was obtained using an automated hemapheresis procedure in a local blood transfusion centre. The platelets obtained from different volunteers were pooled and then divided into aliquots. Each aliquot was subjected to three freeze-thaw cycles to disrupt their membranes and release the growth factors stored in the granules, producing hPLs.

### IGF1 concentrations in platelet lysates

The Human IGF1 ELISA kit (Wuhan Boster Biological Technology LTD, Wuhan, China) was used for the *in vitro* quantitative determination of human IGF1 in FBS (control) and serial dilution of hPL, according to the user’s guide.

### Growth assay

The cells were cultured in 1 % FBS medium containing IGF1 40 ng/ml, the concentration was derived from the IGF1 ELISA dosage in hPL, or hPL corresponding to 3.75 × 10^7^ platelets/ml or equivalent percentage of FBS in presence of 1 μM (HepG2 cells) or 5 μM (Hep3B and PLC/RFP/5) of Regorafenib. In the same growth condition, HCC cells were cultured in absence or presence of IGFR inhibitor, GSK1838705A 1 μM. After defined incubation times, the proliferative response was estimated by colorimetric 3-(4,5 di-methylthiazol-2-yl)-2,5-diphenyltetrazolium bromide (MTT) test. The trypan blue exclusion assay was used to evaluate cell viability. Each experiment was performed in triplicate and repeated three times.

### Cell cycle analysis

PLC/PRF/5 were synchronized by using thymidine 0.2 M added to the medium. After 18 h of incubation, the medium containing thymidine was replaced with fresh medium for 9 h, and then cells were treated with thymidine for an additional 17 h. Cells were separated into two groups: one group was collected for cell cycle analysis and the other one continued culturing; Regorafenib 5 μM, IGF1 40 ng/ml and GSK1838705A 1 μM were added, and after 6 h of treatment cells were collected to be processed, according to the user’s guide, with the Muse Cell Cycle Kit (Millipore, Darmstadt, Germany) which determines the percentage of cells in the G0/G1, S and G2/M phases of cell cycle with the Muse Cell Analyzer.

### Migration assay

A scratch assay was performed as previously described [31, 32]. Briefly, a wound was generated with a pipette tip, after rinsing, medium containing IGF1 40 ng/ml or 1 % FBS (control) alone or in combination with Regorafenib 1 μM and/or GSK1838705A 1 μM. Photographs were taken of each well immediately (T0) and after 24 h (T1), 48 h (T2) and 72 h (T3). The values were expressed as percentage of migration, with 100 % being when the wound was completely closed. The results were representative of three independent experiments.

### Invasion assay

Cell invasion assays were performed using Matrigel (BD Transduction, San Jose, CA, USA)-coated Transwells (8 μm pore PET membrane, Millipore, Billerica, MA, USA) as previously described [31]. Briefly, Regorafenib 5 μM and/or GSK1838705A 1 μM treated cells were suspended in low serum medium. Medium containing IGF 40 ng/ml or FBS was added to the bottom wells. After incubation of 24 h, the invading cells were fixed and stained. The images were acquired and analyzed counting the cells with Image J Software (National Institute of Health, USA). Values obtained were expressed as percentage of invading cells, setting the cell counts of control cells as 100 %. Results were representative of three independent experiments.

### Apoptosis assays - *Annexin V*

The Muse Annexin V/Dead Cell Assay Kit (Millipore, Darmstadt, Germany) for quantitative analysis of live, early/ late apoptotic and dead cells was used with a Muse Cell Analyzer (Millipore). Briefly, the assay utilizes Annexin V to detect PS on the external membrane of apoptotic cells. A dead cell marker (7-AAD) is also used. PLC/PRF/5 cell line, including positive and negative controls, were cultured in 1 % FBS medium in presence of Regorafenib 5 μM alone (control cells) or in combination with IGF1 40 ng/ml or IGF1 40 ng/ml and GSK1838705A 1 μM for 48 h. The cells were then processed as described in the user’s guide.

### Western blots

MAPK signaling and anti-apoptosis markers in cells treated with Regorafenib 5 μM and IGF1 40 ng/mL were analyzed by Western blot, as previously described [31]. Briefly, cells were washed twice with cold PBS and then lysed in RIPA buffer (Sigma-Aldrich, Milan; Italy). After quantization of protein concentration, equal amount of protein (50 μg) were resolved on SDS–PAGE and transferred to polyvinyldifluoride (PVDF) filters. The blots were blocked with 5 % (w/v) nonfat dry milk for 2 h at room temperature and then probed with primary antibody overnight at 4 °C.

The primary antibodies were directed against the following proteins: IGFR-1 and phospho-IGFR-1 (p-IGFR-1), ERK and phospho-ERK (p-ERK), BRAF and phospho-BRAF (p-BRAF), c-Myc and phospho-c-Myc (p-c-Myc), JNK and phospho-JNK (p-JNK), STAT3 and phospho-STAT3 (Tyr705, Ser727) (p-STAT3), p38 and phospho-p38 (p-p38), Bim, Bid, Bad, phospho-survivin (p-survivin), survivin, Bcl-xL, Bcl-2 and β-actin (Cell Signaling, Beverly, MA, USA). After three washes, incubation was followed by the reaction with horseradish peroxidase-conjugated secondary antibody for 1 h at room temperature. The immunoreactive bands were visualized and analyzed using enhanced chemiluminescence detection reagents, according to the manufacturer’s instructions, and chemiluminescence detection system (ChemiDoc XRS apparatus and software, Bio-Rad, Milan, Italy).

### Statistical analysis

GraphPad Prism 5.0 software (La Jolla, CA, USA) was used for all statistical analysis. Mann–Whitney nonparametric test was employed to assess the statistical significance of differences between two groups. For multiple comparisons was used one-way Anova test followed by appropriate post-test. *p* < 0.05 was considered statistically significant. All experiments were done in triplicate and data are presented as mean ± standard deviation (SD).

## Results

### Antagonism by IGF1 of Regorafenib-mediated inhibition of HCC cell growth

hPL were previously examined for the ability to antagonize Regorafenib-mediated inhibition of human HCC cell line growth [29]. To further investigate the role of IGF1, one of the known platelet growth factors, in antagonizing Regorafenib actions on HCC cells, we initially measured IGF1 levels in hPL as described in Materials and Methods. The results showed concentrations of 40 ng/ml of IGF1 were present in hPL, corresponding to 3.75 × 10^7^ platelets/ml. This IGF1 concentration range was used in the subsequent experiments.

Hep3B, PLC/PRF/5 and HepG2 human HCC cell lines were treated in log phase growth in culture dishes with Regorafenib 1–5 μM or IGF1 40 ng/ml alone or in combination with appropriate controls and proliferation was evaluated by MTT assay.

**Fig. 1:**
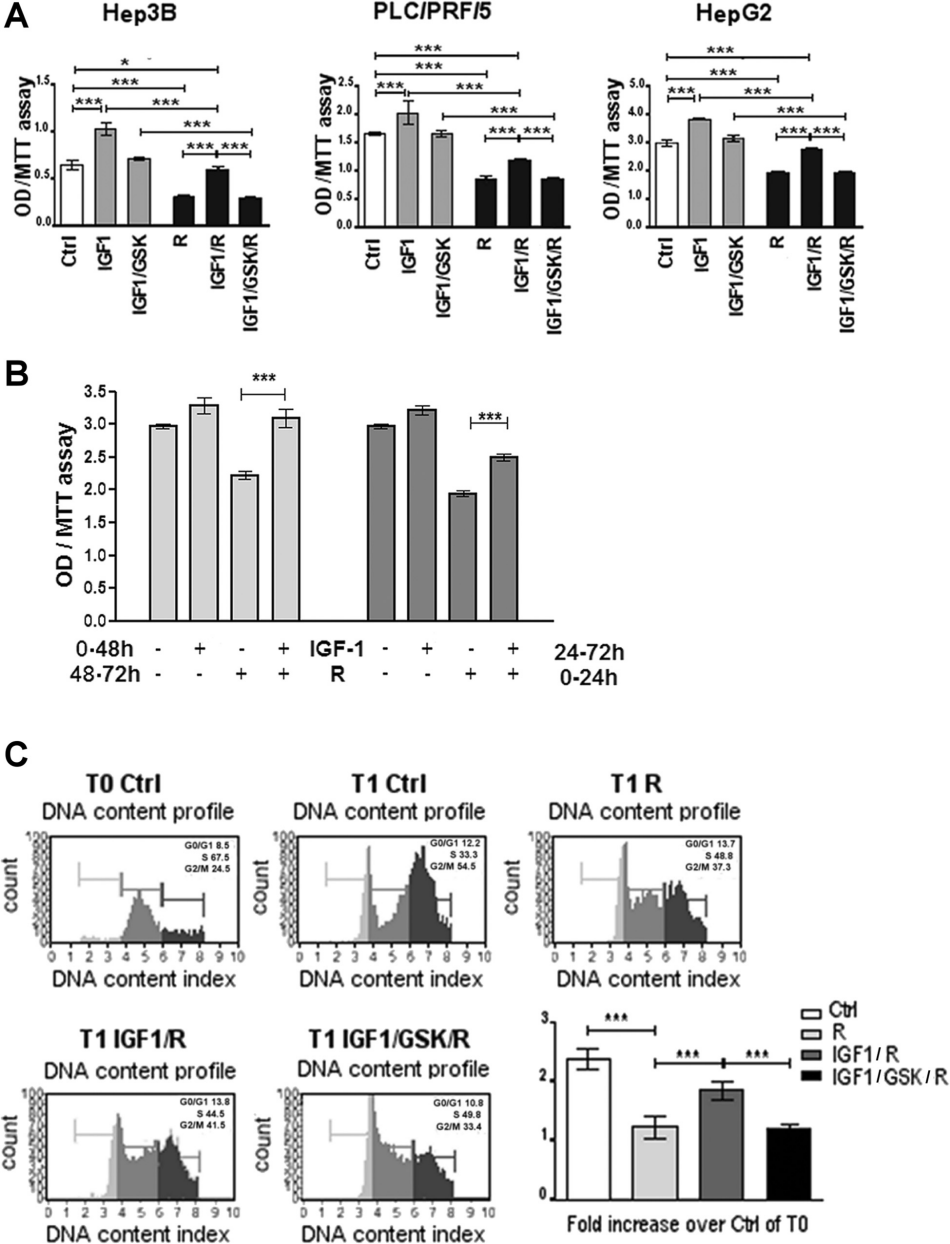
Antagonism by IGF1 of Regorafenib-mediated growth inhibition of HCC cell Lines. **a**. Hep3B, PLC/PRF/5 and HepG2 cells were cultured in presence of Regorafenib 1–5 μM, IGF1 40 ng/ml, GSK1838705A 1 μM. MTT assay was assessed after 48 h (48 h). **b**. PLC/PRF/5 cells were cultured in presence of Regorafenib 5 μM and IGF1 40 ng/ml in different time conditions. In the first group (light gray) the cells previously treated with IGF1 for 48 h received Regorafenib for the following 24 h. In the second group (dark gray) the cells, previously treated with Regorafenib for 24 h, received IGF for the following 48 h. MTT assay was assessed after 72 h. **c**. PLC/PRF/5 cells were synchronized in the S phase of the cell cycle using thymidine 0.2 M (T0), after 6 h from block release (T1), the cells were processed with the Cell Cycle Kit and analyzed with Muse Cell Analyzer to evaluate the percentage of cells in G0/G1, S and G2/M phases. The panels represent an example of DNA content profile in different treatment conditions. The mean of three independent experiments was plotted in the relative graph, where the values are calculated as fold increase of the cells in G2/M at T1 respect to control cells at T0. The results of three independent experiments are expressed as means ± SD. ***p < 0.0001

We found that IGF1 significantly antagonized the growth inhibitory actions of Regorafenib. We found that in all HCC cell lines that were examined, IGF1, added in combination with Regorafenib, significantly increased the proliferation rate of about 60 %, compared to the same cells treated only with Regorafenib. This effect was only partial because the proliferation rate of IGF1/R treated cells was still lower than both IGF1 (42 %) and control (15 %) cells and was blocked by GSK1838705A, a potent inhibitor of IGF1 receptor, used at a non- toxic concentration (1 μM) that did not by itself affect proliferation (Fig. 1a).

We next investigated if the timing of the IGF1 addition might affect Regorafenib-mediated growth inhibition. For this purpose, two different culture conditions were used. In the first condition, cells that had been previously cultured for 48 h with IGF1 40 ng/ml or equivalent percentage of FBS (control) were then treated with Regorafenib 5 μM for the next 24 h. In the second condition, cells that had been pre-treated for 24 h with Regorafenib were subsequently cultured for the next 48 h in the presence of IGF1 or FBS. In Regorafenib-treated cells, IGF1 pretreatment led to an increase in the proliferation rate (40 %) compared to the proliferation rate observed in cells treated only with drug (Fig. 1b). Moreover, IGF1 partially reversed (28 %) the Regorafenib-mediated growth inhibition if added after drug administration. These results confirmed our previous findings in which the reversible effect of Regorafenib was shown [33].

The antagonism exerted by IGF1 on Regorafenib-mediated growth inhibitory actions was also observed on cell cycle progression. Regorafenib caused an inhibition in the progression from S phase of the cell cycle to G2/M phase. After 6 h (T1) from block release (T0), Regorafenib treated cells in G2/M phase were only 0.2 times more than the control cells at T0, while the number of control cells in G2/M phase at T1 were 1.5 times more with respect the number of control cells at T0 (Fig. 1c). IGF1 counteracted the Regorafenib-mediated block in cell cycle progression (0.85 times more than the control cells at T0), and the IGFR antagonist, GSK1838705A, abrogated this effect.

### Antagonism by IGF1 of Regorafenib-mediated induction of apoptosis

**Fig. 2:**
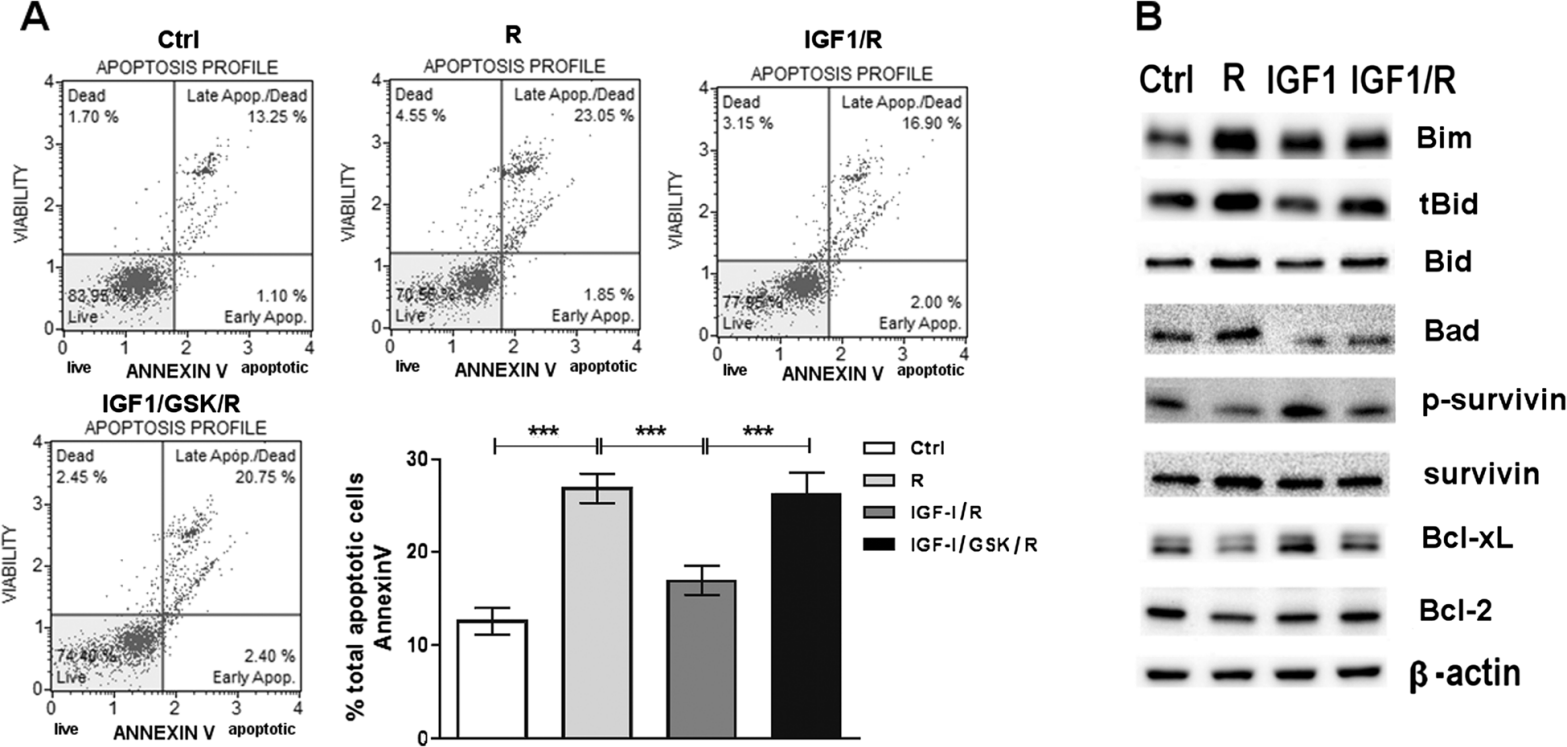
Antagonism by IGF1 of Regorafenib-mediated induction of apoptosis. **a**. PLC/PRF/5 cell line cultured in 1 % FBS medium was treated with IGF1 40 ng/ml in combination with Regorafenib 5 μM and GSK 1 μM. The Muse Annexin V kit was used to evaluate the percentage of apoptotic cells. The means ± SD of three independent experiments is plotted in the relative graph. ***p < 0.0001. **b**. Representative Western blot that shows anti-apoptotic (phospho-survivin, Bcl-xL and Bcl-2) and pro-apoptotic (Bim, truncated-Bid and Bad) proteins in PLC/PRF/5 cells treated as described above

The effects of IGF1 on Regorafenib–mediated apoptosis, a major aspect of its growth-inhibitory effects, were then examined. Regorafenib induced an increase in cellular Annexin V. When IGF1 was also added to the cell medium together with Regorafenib, a pronounced and significant antagonism of apoptosis induction was found and this antagonism was abrogated by the IGFR inhibitor GSK1838705A 1 μM (Fig. 2a). Major apoptosis markers in cells treated with Regorafenib alone or in combination with IGF1 were then examined. We found that pro-apoptotic marker (Bim, tBid and Bad) levels increased in the presence of Regorafenib alone and anti-apoptotic markers (p-survivin, Bcl-xL and Bcl-2) decreased under the same conditions. However, in cells treated with Regorafenib in combination with IGF1 (Fig. 2b), we found that IGF1 antagonized these Regorafenib effects on induction of apoptosis.

### Antagonism by IGF-1 of Regorafenib-mediated inhibition of cell migration and invasion

**Fig. 3:**
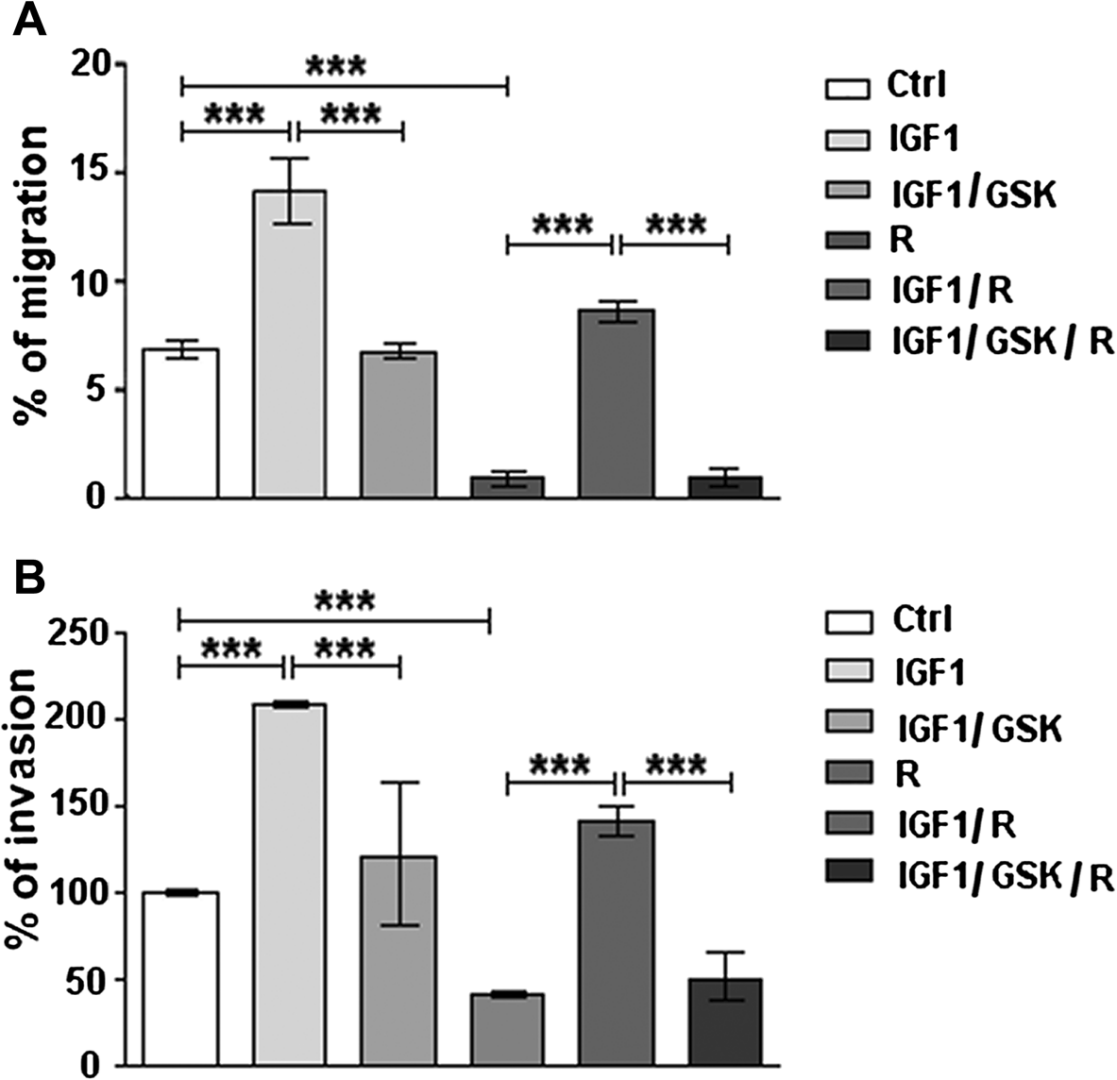
Antagonism by IGF1 of regorafenib-mediated inhibition of migration and invasion. PLC/PRF/5 cell line cultured in 1 % FBS medium was treated with IGF1 40 ng/ml alone or in combination with Regorafenib 5 μM and GSK 1 μM. **a**. Migration assay was performed as described and the microscopic analysis was assessed at the time of the scratch (T0) and after 48 h (T2). The values were expressed as percentage of migration, where 100 % represents the scratch completely closed. **b**. The percentage of invasion was calculated comparing the invading drug-treated cells to drug-untreated control cells (100 %). The results of three independent experiments are expressed as means ± SD. ***p < 0.0001

Regorafenib inhibits both HCC cell migration, as well as cell invasion through Matrigel membranes [31]. IGF1 40 ng/ml was then added to cells in the presence of Regorafenib 5 μM, a concentration that can inhibit both migration and invasion in HCC cells. We found that IGF1 antagonized the inhibition by Regorafenib of both migration and invasion and these effects were also abrogated by GSK1838705A 1 μM (Fig. 3a/b).

**Fig. 4:**
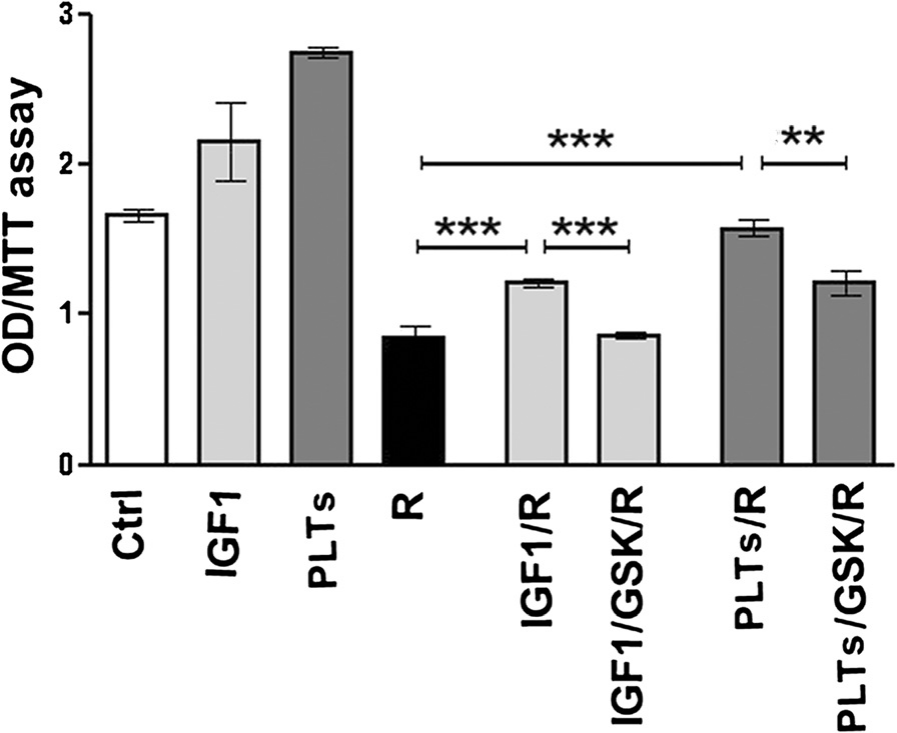
Effects of IGF1 receptor (IGFR1) inhibitor (GSK1838705A) on Regorafenib-mediated growth inhibition in PLC/PRF/5 cells. PLC/PRF/5 cells were cultured in 1 % FBS medium in presence of IGF1 40 ng/ml or platelet lysate corresponding to 3.75 × 10^7^ platelets/ml, Regorafenib 5 μM and GSK1838705A 1 μM, using the conditions indicated in the graph. MTT assay was assessed after 48 h. The results of three independent experiments are expressed as mean ± SD. **p < 0.001; ***p < 0.0001

### IGF-1 as a major contributor to hPL actions

The above results using GSK1838705A, showed that IGF1 receptors were involved in the mechanisms of IGF1-mediated antagonism of Regorafenib effects. We then examined the possibility that the blocking effects of hPL on Regorafenib that had been previously noted, might be attributable mostly to IGF1 presence in platelet lysates [28]. We found that the platelet lysates increased the proliferative rate of Regorafenib-treated cells by 84 %. This effect was attributed to IGF1 action for 41 %, as demonstrated adding GSK1838705A 1 μM to the platelet lysates, and the remaining 43 % was due to the combined effects exerted by all the other growth factors included in platelets (Fig.4).

### Effects of IGF1 on cell signaling

**Fig. 5:**
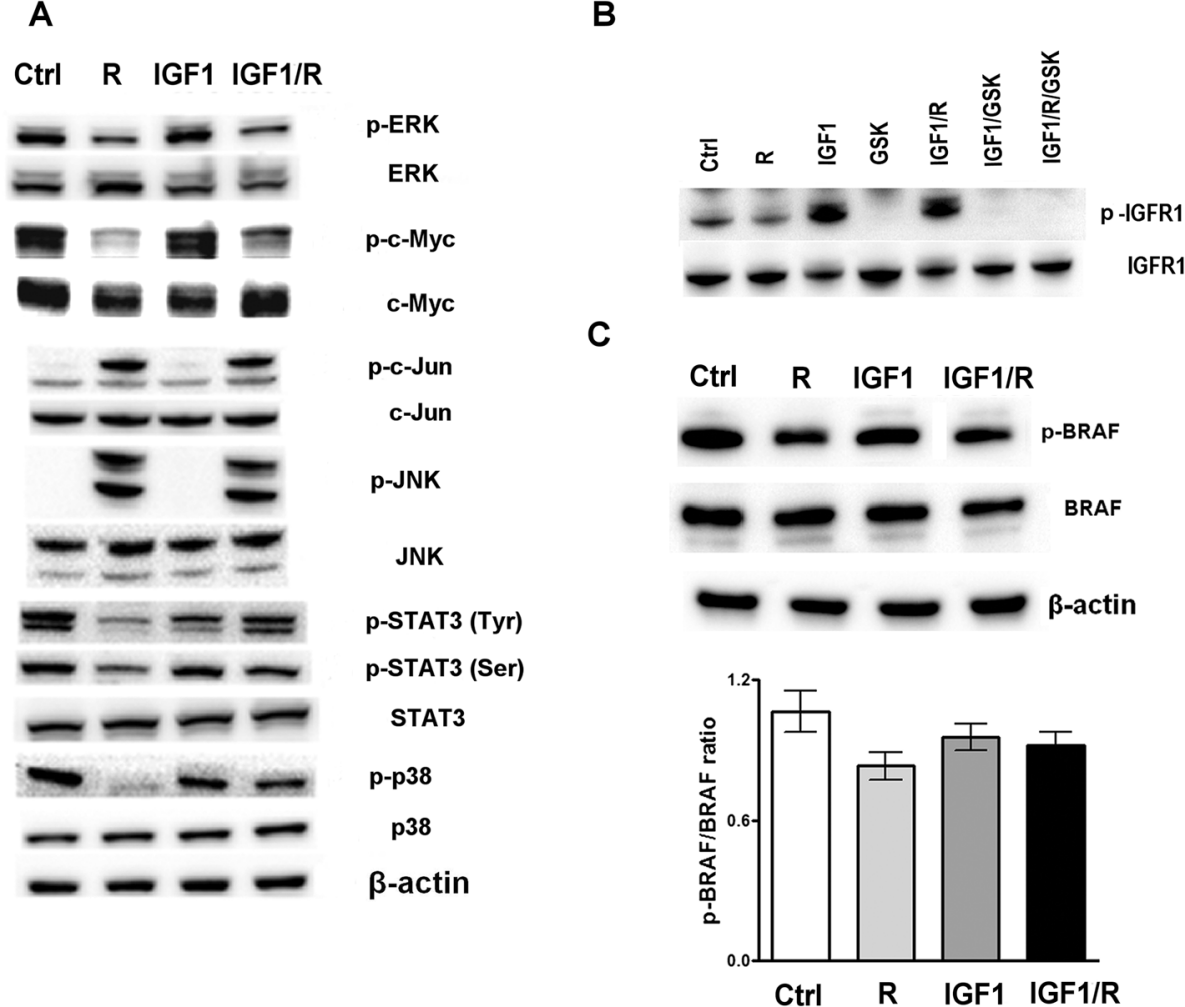
MAPK signaling and IGFR1 expression in PLC/PRF/5 cells. **a**. Western blot of MAPK proteins that shows IGF1 antagonism effect on Regorafenib action in PLC/PRF/5 cells. **b**. Activated IGF1 receptor (IGFR1) after treatment with Regorafenib (R), IGF1, IGF1 + R, IGF1 + GSK and IGF1 + R + GSK, respectively. **c**. IGF1 effect on the BRAF protein that is direct Regorafenib target

Regorafenib has previously been shown [28] to cause a decrease in p-ERK levels, consequent on Raf inhibition. We next examined the effects of Regorafenib on cellular MAPK signaling pathway marker levels in cells that were treated in the absence or presence of IGF1. We found that IGF1 antagonized the Regorafenib-mediated decrease in p-BRAF and p-ERK levels, and also antagonized the decreases in the levels of p-c-Myc, p-p38 and p-STAT3 (Tyr705, Ser727). By contrast, the Regorafenib-mediated increases in p-JNK levels were decreased by the addition of IGF1 to the cultures (Fig. 5a). These actions of IGF1 were in turn blocked by the presence in the cultures of GSK1838705A, indicating the involvement of the IGF1 receptors. IGF1 thus counteracted Regorafenib actions on signaling intracellular markers (Fig. 5b/c).

## Discussion

Platelets and their contained growth factors have become of great interest in tumor biology, due to their contribution to the tumor microenvironment, their content of many growth and inflammatory factors, and to their identification as important contributors to tumor growth and metastasis, including HCC, as well as to normal liver regeneration [21, 22, 34–36]. Amongst the many factors in platelet granules, IGF1 has received considerable attention, due to its involvement in HCC growth and prognosis [37–39] and as a possible target of anti-cancer and anti-HCC therapies.

Regorafenib is a multikinase inhibitor and its action is similar of Sorafenib [40]. Sorafenib is a small multi-target inhibitor that has broad-spectrum activity against several tyrosine kinases. Although initially described as a b-Raf inhibitor, Sorafenib inhibits the activity of several cell surface receptor tyrosine kinases [40]. A constitutive activation of receptor tyrosine kinases is a common feature of many types of cancers, particularly those of soft tissue origin where their concerted expression has been shown to promote tumor growth and survival [41]. Regorafenib blocks ERK action, an intracellular signaling involved in HCC cell proliferation [32].

Also Regorafenib has an indirect effect of p38 kinase, JNK and Stat3 involved in tumorigenesis and HCC proliferation. In this study we explored the action of IGF1 in HCC cells *in vitro*. We found that IGF1 antagonized the growth inhibitory effects of Regorafenib. The insulin-like growth factor (IGF) pathway has highly conserved functions in mammals and plays a critical role in energy metabolism and cell renewal in response to nutrients. IGF pathway is not only involved in cell growth in tissue culture, but it also promotes cell proliferation, migration and transformation into malignancy [42]. The anti-apoptotic property of IGF-1R was shown in its response to p53, the tumor suppressor gene that promotes apoptosis [42]. Wild type p53 expression inhibited the gene expression of IGF-1R, while mutant p53 increased the gene expression of IGF-1R. Oncogenes such as Src kinase and Akt kinase both stimulated the gene expression of IGF-1R, providing more evidence that IGF-1R is vital in carcinogenesis [43]. In addition, IGF-1R also stimulates cell mobility, as demonstrated by its activity in melanoma cell lines. Another important role of IGF-1R in carcinogenesis is its ability to transform and maintain the transformed phenotype [42]. IGF1 and its binding to its receptor induce activations of two major intracellular cascades, the phosphatidyl inositol 3-kinase (PI3K) and the mitogen-activated protein kinase (MAPK), both of which result in cell differentiation, proliferation and anti-apoptosis [43]. Particularly in HCC, IGF-1R is overexpressed and can induce carcinogenesis. In a study where 10 HCC cell lines (including PLC/PRF/5 cell line) were tested, all of them showed elevated IGF-1R mRNA. Furthermore, the addition of IGF-1 to the PLC/PRF/5 cell line induced increased cell proliferation in a dose dependent manner, showing that the major tumor promoting effects of IGF ligands on HCC are exerted through IGF-1R [42–44].

The IGF1 signaling pathway provides an important regulatory mechanism for tumorigenesis and drug resistance in HCC [41].

We found that IGF1, used in the same concentration as was measured in hPL, significantly antagonized the growth inhibitory actions of Regorafenib. This effect was blocked by GSK1838705A, a potent inhibitor of IGF1 receptors, used at non-toxic concentrations that do not affect proliferation. We next found that IGF pre-treatment protected the cells from subsequent addition of Regorafenib to the cultures and antagonized Regorafenib-mediated growth inhibition, which was reduced by 40 % when the cells were pre-treated with IGF1. This suggested that IGF1 signalling is implicated in the observed Regorafenib resistance or that a major mechanism of Regorafenib-mediated growth control is exerted through interference in the IGFR pathway, which is then counteracted by addition of IGF1 [42]. Moreover, the Regorafenib pre-treatment modified the stimulatory action ensuing to the addition of the IGF1. Regorafenib-mediated inhibition of cell growth was only partially rescued by subsequent IGF1 treatment (28 %), showing that Regorafenib treatment, even if reversible, modified the cells [43].

A corollary is that IGFR inhibitors might enhance the growth inhibitory actions of Regorafenib, since IGF1 can block the drug effects on cell growth. Our results on cell cycle progression support the idea of antagonism exerted by IGF on Regorafenib-mediated growth inhibition.

An important mechanism of resistance to IGFR inhibitors is the compensatory activation of related signalling pathways [44]. We observed that IGF1 changed intracellular signalling in HCC cells. We found that levels of the proliferation markers p-BRAF, p-ERK, p-p38 and p-Stat3 (Tyr705, Ser727) were decreased by Regorafenib action. By contrast, IGF1 addition to the cultures antagonized Regorafenib action and levels of the proliferation markers increased. We, also, found that levels of apoptosis markers were influenced by IGF1 actions, since pro-apoptotic markers (p-JNK, p-c-Jun, Bim, Bad and Bid) decreased with IGF1, while anti-apoptosis markers (survivin, BCL-xL and Bcl-2) increased. Survivin is an apoptosis-inhibitory protein that is over-expressed in multiple cancer types, including HCC, plays critical roles in regulating apoptosis, cell proliferation and survival and has been shown to be a direct downstream target of IGF1 pathway [44].

Regorafenib decreased p-survivin levels and this was also antagonized by addition of IGF1. GSK1838705A reversed the IGF1 actions, showing that the antagonist effects of IGF are exerted through its receptor. The antagonism exerted by IGF on Regorafenib effects was also shown for drug mediated apoptosis, as a decrease in Annexin V levels and in the Caspase 3/7 activation. Pre-clinical studies have shown that the efficacy in anti-cancer therapy for HCC can be improved by inhibiting the IGF signalling pathway in HCC cells [45].

Several new inhibitors of IGF1 or its receptor are in current clinical trials for HCC, including Octreotide (Novartis), a Somatostatin analog and MEDI 573 (Astrazeneca), targeting either IGF1 and or IGFII, as well as Linsitinib (OSI Pharmaceuticals), Cixutumumab (ImClone), AVE1642 (Sanofi-Aventis), which are IGF receptor antagonists. However, the relationship of IGF1 levels in serum and tumor tissue and of its receptor levels with HCC growth and prognosis are very complex [37]. It has recently been shown that one of the many mechanisms of Sorafenib (Regorafenib is Fluoro-Sorafenib) action on HCC cell growth is via an inhibition of IGF1 [39]. Furthermore, IGF1 has been shown to be involved in resistance to cytotoxic chemotherapy by several drugs and on a variety of tumor types, mainly through an anti-apoptotic effect [33, 46–51], as we have found in the current work.

## Conclusion

These experiments highlight the importance of the microenvironment, including IGF1, in modulating the growth inhibitory effects of anti-HCC therapeutic drugs. By contrast, anti-IGF or anti-IGF receptor agents might be predicted to be therapeutically useful in enhancing the activity of anti-HCC therapies. Finally, we re-enforce the concept of drug resistance mediated by factors in the tumor microenvironment, and also summarize potential drug targets based on the current knowledge of the tumor microenvironment [52].

## Abbreviations

HCC: Hepatocellular carcinoma
hPL: Human platelet lysates
ERK: Extracellular signal-regulated kinase
JNK: c-Jun NH2-terminal kinase
STAT: Signal transducer and activator of transcription-3
WB: Western blot
MTT: 3-(4,5-Dimethylthiazol-2-yl)-2,5-diphenyltetrazolium bromide
BRdU: BrdU 5-bromo-2’-deoxy-uridine
IGF1: Insulin like growth factor 1
